# Global seaweed productivity

**DOI:** 10.1126/sciadv.abn2465

**Published:** 2022-09-14

**Authors:** Albert Pessarrodona, Jorge Assis, Karen Filbee-Dexter, Michael T. Burrows, Jean-Pierre Gattuso, Carlos M. Duarte, Dorte Krause-Jensen, Pippa J. Moore, Dan A. Smale, Thomas Wernberg

**Affiliations:** ^1^ UWA Oceans Institute and School of Biological Sciences, University of Western Australia, Crawley, WA 6009, Australia.; ^2^ CCMAR, CIMAR, Universidade do Algarve, Gambelas, Faro, Portugal.; ^3^ Institute of Marine Research, His, Norway.; ^4^ Scottish Association for Marine Science, Oban, Argyll PA37 1QA, UK.; ^5^ CNRS, Laboratoire d’Océanographie de Villefranche, Sorbonne Université, 181 chemin du Lazaret, F-06230 Villefranche-sur-mer, France.; ^6^ Institute for Sustainable Development and International Relations, Sciences Po, 27 rue Saint Guillaume, F-75007 Paris, France.; ^7^ Red Sea Research Centre (RSRC) and Computational Bioscience Research Center (CBRC), King Abdullah University of Science and Technology, Thuwal 23955, Saudi Arabia.; ^8^ Arctic Research Centre, Aarhus University, Aarhus C, Denmark.; ^9^ Department of Ecoscience, Aarhus University, Vejlsøvej 25, DK-8600 Silkeborg, Denmark.; ^10^ The Dove Marine Laboratory, School of Natural and Environmental Science, Newcastle University, Newcastle upon Tyne NE1 7RU, UK.; ^11^ Marine Biological Association of the United Kingdom, Citadel Hill, Plymouth PL1 2PB, UK.; ^12^ Roskilde University, Box 260, 4000 Roskilde, Denmark.

## Abstract

The magnitude and distribution of net primary production (NPP) in the coastal ocean remains poorly constrained, particularly for shallow marine vegetation. Here, using a compilation of in situ annual NPP measurements across >400 sites in 72 geographic ecoregions, we provide global predictions of the productivity of seaweed habitats, which form the largest vegetated coastal biome on the planet. We find that seaweed NPP is strongly coupled to climatic variables, peaks at temperate latitudes, and is dominated by forests of large brown seaweeds. Seaweed forests exhibit exceptionally high per-area production rates (a global average of 656 and 1711 gC m^−2^ year^−1^ in the subtidal and intertidal, respectively), being up to 10 times higher than coastal phytoplankton in temperate and polar seas. Our results show that seaweed NPP is a strong driver of production in the coastal ocean and call for its integration in the oceanic carbon cycle, where it has traditionally been overlooked.

## Teaser

Global estimates show that seaweed habitats are a strong driver of productivity in the coastal ocean.

## INTRODUCTION

Net primary production (NPP) is a major driver of the carbon cycling through the biosphere. Roughly half of the planet’s NPP occurs in the ocean (*1*), and quantifying the sources, patterns, and drivers of NPP is therefore a fundamental goal of biological oceanography. While the advent of remote sensing technology has facilitated the measurement of NPP over unprecedented scales in the open ocean (*2*), the productivity of the coastal ocean—where remotely sensed measurements are often challenged (*3*, *4*)—remains more poorly constrained. That is particularly true for shallow coastal fringes, where marine vegetation such as seagrass beds or seaweed forests can contribute significantly to primary production (*5*–*7*), but whose global NPP patterns are still unresolved. As a result, the quantitative importance of marine vegetation carbon fixation relative to that of other primary producers (e.g., phytoplankton) is largely unknown, leading to coastal carbon fluxes and processes being inadequately represented in the global carbon budget and Earth system models (*8*). This includes the NPP of seaweeds, which form the largest and most productive coastal vegetated biome, drawing an annual global CO2 flux comparable to that of the Amazon rainforest (*9*). Seaweed productivity represents a key source of carbon to nearshore food webs but also to the open ocean (*10*), where it can support food webs and carbon burial (*11*).

Despite the immense carbon fixation capacity of seaweeds and their contribution to carbon cycling having been known for decades (*12*–*14*), global assessments of the distribution and determinants of their NPP remain elusive. As seaweed NPP has traditionally been measured in situ, measurements are usually restricted to easily accessible locations, with the seaweed productivity of much of the world’s coastlines remaining virtually unknown. This knowledge gap is a known source of error in the current estimates of global seaweed NPP, which range 20-fold depending on the number of locations and estimates used (*9*, *15*–*17*). Current global estimates also do not account for the different techniques used to measure NPP, which are known to capture different aspects of NPP (*18*), or NPP variability between different taxonomic groups, further contributing uncertainty (*15*). Quantifying the NPP of different seaweed groups across their global extent thus remains a key barrier to reliably resolve the contribution of seaweed to oceanic carbon cycles.

Environmental factors such as temperature and photosynthetically active radiation (PAR) strongly shape the NPP of seaweed vegetation (*19*, *20*) and can reliably predict NPP over seasons spanning large variability in climatic forcing (*21*). A promising method to quantify the global distribution of seaweed productivity is thus to develop mechanistic models linking NPP with its climatic forcing variables. This approach is already widely used to estimate terrestrial primary production (*22*), and available evidence suggests that it can be applied for seaweed vegetation (*21*). However, efforts to date have so far been hampered by the lack of (i) data-driven models scaling up area-based NPP at regional and global levels and (ii) a global dataset of NPP observations to ground-truth estimates on. Here, we collate the most comprehensive dataset of wild seaweed NPP measurements to date and model area-based seaweed productivity rates across the coastal ocean. Compiled NPP measurements cover more than 400 unique sampling sites distributed across all ocean realms (fig. S1), spanning seaweed habitats from pole to pole and from the high-tide mark to depths of >50 m. Our dataset (*23*) quantifies net carbon production on a yearly per-area basis (i.e., gC m^−2^ year^−1^) and contains information on more than 240 species or taxonomic entities, encompassing all major seaweed clades and functional forms. We use this dataset to model and map area-based seaweed NPP, yielding the first predictions of the geographical distribution of NPP for any vegetated habitat in the coastal ocean. Our predictions focus on algal turfs and seaweed forests formed by large brown algae, as the limited data available on other vegetation types (e.g., coralline algae and rhodolith beds and other algal beds) precluded meaningful global estimates. Available evidence suggests, however, that algal turfs and seaweed forests are responsible for more than 70% of the total carbon assimilated by seaweeds globally (*9*, *16*) and therefore are the most important drivers of global seaweed productivity. Last, we use these predictions to spatially quantify the relative importance of seaweed productivity with that of coastal phytoplankton and compare its patterns to that of other primary producers on Earth.

## RESULTS

### Patterns of observed seaweed NPP across groups, taxa, and methodology

Compilation of NPP records revealed that seaweed NPP varied by several orders of magnitude (<0.01 to 5000 gC m^−2^ year^−1^) but exhibited clear patterns across vegetation types, methodology, and latitude. Seaweed forests, habitats dominated by canopies of tall brown algae, had the highest average NPP of any seaweed habitat, followed by pelagic blooming algae (e.g., “green tides” of *Ulva* spp.) and algal turfs—low-lying aggregations of single or multiple species of algae common in tropical reefs and temperate areas (Table 1). The highest NPP rates were documented in *Durvillaea* spp., whose standing forest biomass can reach upward of 100 kg fresh weight m^−2^ (*24*), but are largely restricted to intertidal areas. Floating pelagic *Sargassum* mats, despite forming extensive blooms, had the lowest NPP rates.

**Table 1.** Summary of observed seaweed NPP rates by vegetation type. Categories were based on vegetation height, dominant vegetation (brown, red, or green algae), and their position in the water column (benthic or pelagic). Values are means ± SE, while the number of measurements in each habitat type is indicated in parenthesis. Letters denote significant pairwise NPP differences between vegetation types in a linear model fitted with a gamma distribution.

**Vegetation type**	**Description**	**Examples**	**NPP (gC m^−2^ year^−1^)**
Seaweed forest	Vegetation dominated by tall canopies of brown algae from the orders Laminariales, Fucales, Tilopteridales, and Desmarestiales. Includes understory and epiphytic taxa associated with the canopies.	Kelp and *Sargassum* forests	536 ± 31 (518)^A^
Algal turfs	Low-lying vegetation dominated by aggregations of single or multiple species of short algae from different groups, forming a complex matrix	Algal turfs	321 ± 22 (118)^B^
Brown algal beds	Low-lying vegetation dominated by brown algae	*Padina* and *Dictyota* beds	166 ± 97 (7)^AB^
Red algal beds	Low-lying vegetation dominated by red algae	*Gelidium* and *Gracilaria* beds	194 ± 23 (73)^B^
Green algal beds	Low-lying vegetation dominated by green algae	*Caulerpa* beds and *Halimeda* bioherms	134 ± 43 (70)^B^
Rhodolith beds and coralline algae	Habitats of coralline algae and rhodolith beds	Coralline barrens, trottoir, and rhodolith beds	207 ± 44 (17)^AB^
Floating *Sargassum*	Pelagic *Sargassum* rafts	*Sargassum fluitans* and *Sargassum natans*	0.2 ± 0.1 (5)^B^
Other floating algae	Other free-floating aggregations of algae on the bottom or at the sea surface	*Ulva* blooms	394 ± 112 (7)^AB^

Measured NPP per unit area peaked in temperate latitudes (30°S to 50°S and 40°N to 60°N), decreasing toward the tropics and polar regions (fig. S2). Latitudinal patterns were consistent irrespective of the methodology used to estimate NPP (fig. S2), although, as expected (*18*), photorespirometry-based methods generally yielded higher estimates than those based on plant biomass accumulation (fig. S3 and table S1).

### Modeled seaweed NPP

We then modeled the productivity of seaweed forests and algal turfs across the world’s coastlines where they occur, as information regarding the global distribution of other seaweed vegetation types (cf. Table 1) was lacking or measurements of their NPP were limited (*n* < 100). These were modeled separately as turfs often occupy different areas within a reef and are composed of algal functional forms with different photosynthetic performance than forest-forming seaweeds (*25*). We also constructed separate models for intertidal and subtidal seaweed forests (84 and 221 sites, respectively), as environmental variables mediate NPP differently across the intertidal-subtidal boundary (*26*). For algal turfs, we only modeled productivity in the subtidal (107 sites) as intertidal NPP measurements were absent.

**Fig. 1.**
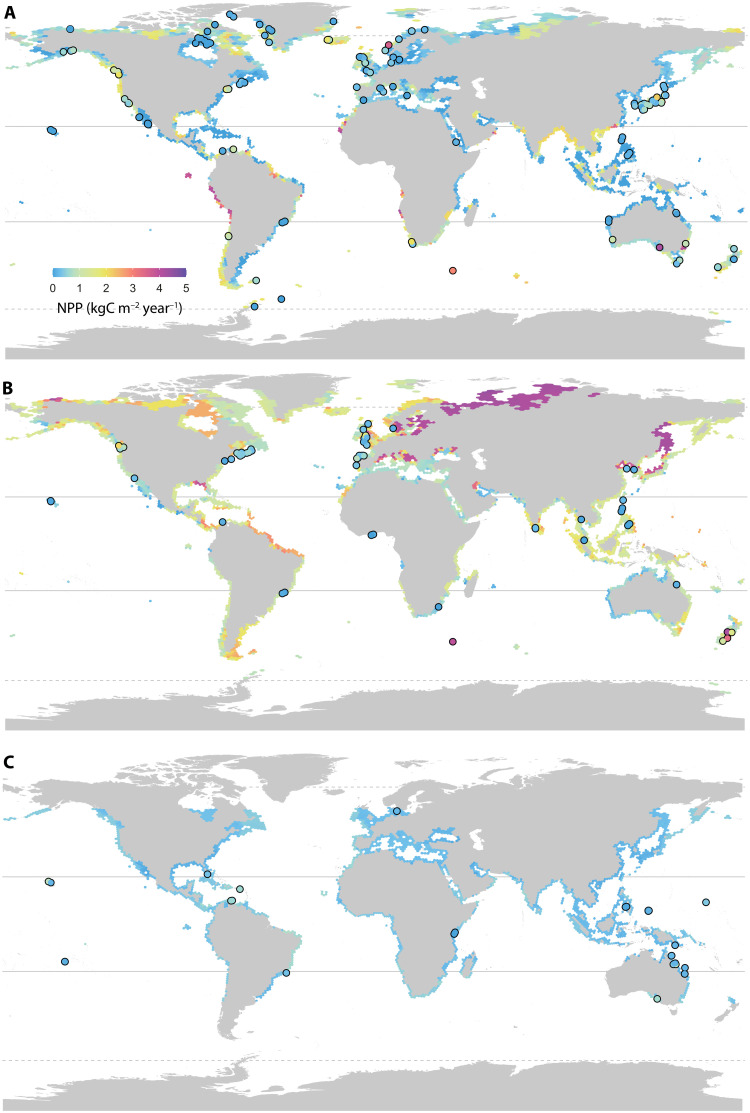
Global distribution of NPP from seaweed vegetation. Globally predicted NPP of subtidal (**A**) and intertidal (**B**) seaweed forests dominated by large brown canopy-forming algae and (**C**) subtidal algal turfs. Points show the location of the study sites included in our database (raw NPP is indicated by the colored dots). Lines depict the tropics (straight) and polar circles (dashed).

Modeled productivities across the global coastlines where seaweed forests and algal turfs occur showed distinct patterns depending on the vegetation type, with similar trends to the raw data. The global NPP of subtidal and intertidal seaweed forests averaged 656 and 1711 gC m^−2^ year^−1^, respectively, and showed marked variation across latitude (range, 0 to 4768 gC m^−2^ year^−1^; Fig. 1, A and B). Algal turfs had lower average productivity (344 gC m^−2^ year^−1^) and showed less variation in predicted NPP (range, 0 to 829 gC m^−2^ year^−1^; Fig. 1C). Overall, our models performed well, with little to moderate deviation between observations and predictions (Fig. 1, 0.63 to 0.91 deviance explained; fig. S4). Globally, seaweed forests were on average ~2 to 3 times more productive per unit area than oceanic phytoplankton, exhibiting rates comparable or higher than most terrestrial ecosystems and agricultural crops (table S2).

The global maps presented here were mostly informed by measurements based on biomass accumulation, as that is the most common methodology used to measure NPP [83% of records; see the Supplementary Materials and (*23*)]. SD around models averaging individual predictions based on photorespirometry or biomass accumulation measurements was largest in areas with high predicted NPP (e.g., Humboldt upwelling), as well as coastlines where little or no NPP measurements were available (e.g., Arctic, Amazon basin, Bay of Bengal, and South China Sea) (fig. S5). This suggests that NPP may not have been fully captured by biomass-based methods in areas of high potential productivity and/or that model predictions overestimated NPP in certain climate envelopes where seaweed NPP has yet to be measured. Biomass accumulation methods only measure carbon allocated to tissue growth and do not include processes incurring carbon losses such as exudation of dissolved organic carbon, particulate carbon export, or herbivory (*18*). A review of seaweed carbon exudation rates showed that the highest rates were recorded in areas producing >5000 gC m^−2^ year^−1^ (*27*), although they could account between 8 and 80% of the total fixed carbon alone (16 to 4400 gC m^−2^ year^−1^; fig. S6). It is possible then that biomass accumulation–based predictions are underestimating true NPP in areas of high productivity; however, we could not adjust them for dissolved carbon exudation rates as data are scarce (11 assays) and have large inconsistencies in methodology among studies. This typically unmeasured—but potentially substantial—carbon flux suggests that the contribution of seaweeds to the oceanic carbon cycle may be substantially underestimated. Caution should, however, be taken when interpreting values from highly productive regions until further investigations in those areas are conducted.

**Fig. 2.**
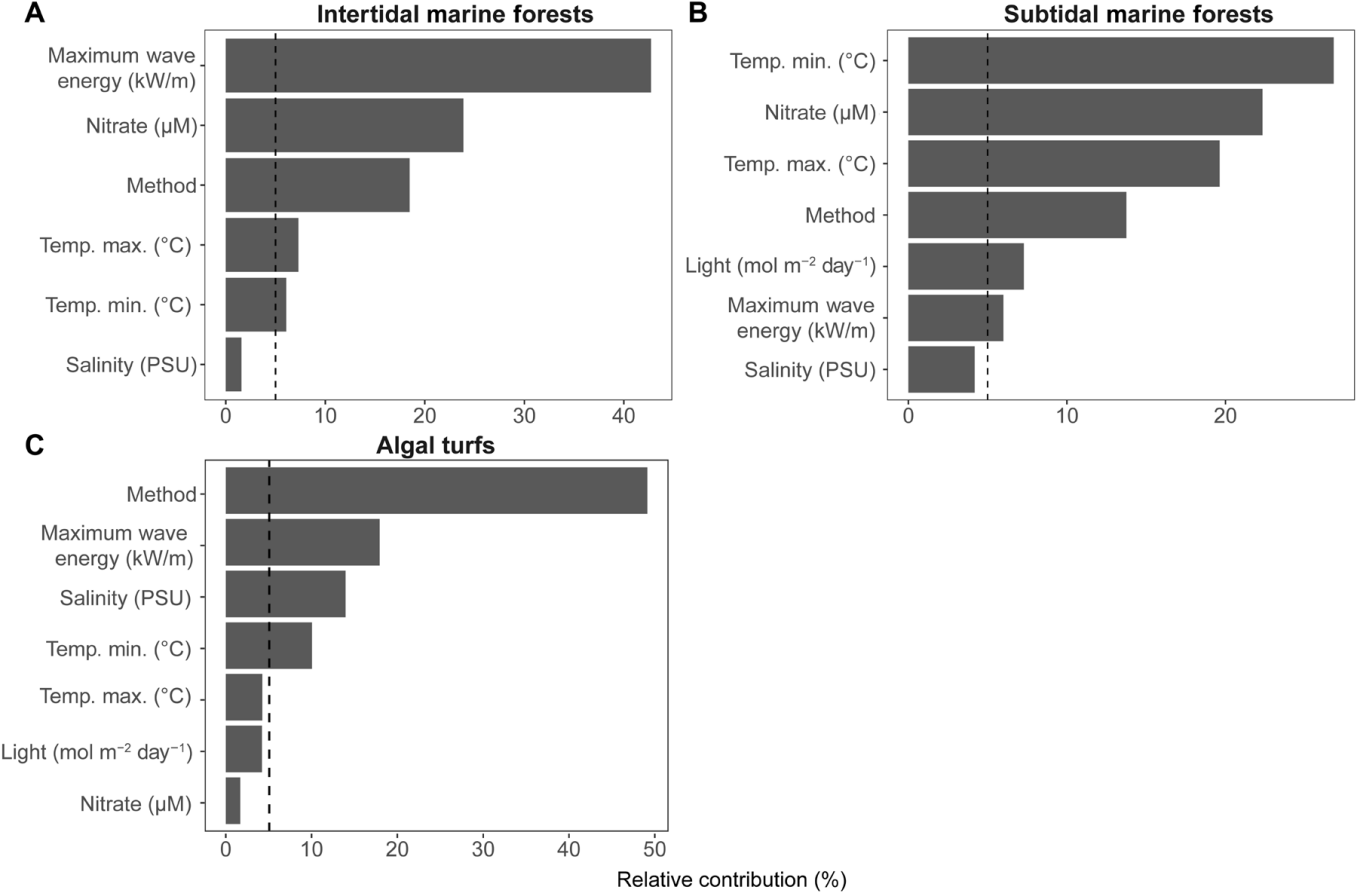
Relative contribution (%) and threshold of climate and methodological variables used to predict NPP. (**A**) Intertidal and (**B**) subtidal seaweed forests formed by large brown algae and (**C**) algal turfs. Dashed lines depict contributions >5%. Note that light was not included as a predictor for the intertidal seaweed forest model. PSU, Practical Salinity Units.

Key predictors in our models were temperature, maximum wave energy (an indicator of coastal exposure), nutrients, and light (PAR) (Fig. 2)—which are all known to limit seaweed growth in different areas of the planet (*20*, *28*, *29*). The methodology used to estimate NPP was also an important variable in all models and particularly so for algal turf habitats where it was the top predictor. Globally, productivity peaked at intermediate annual mean temperatures (10° to 18°C), intermediate irradiances (30 to 40 mol photons m^−2^ day^−1^), and nitrate concentrations >1 μM (fig. S7). These conditions are mostly found in temperate regions, which, consistent with our predictions, also featured the highest measurements of seaweed standing biomass (*30*). Seaweed forests in sub-Antarctic islands and temperate South Africa and South America were predicted to have the largest average NPP (777 to 1758 gC m^−2^ year^−1^; table S3). There was, however, heterogeneity in productivity within temperate latitudes, with high regional NPP predicted near eastern boundary upwelling systems (e.g., Humboldt, Benguela, and Northwest Africa) and low regional NPP in several marginal seas (e.g., Eastern Mediterranean Sea, Baltic Sea, Gulf of California, and Sea of Okhotsk), which typically feature oligotrophic conditions and/or low salinity.

Tropical regions were predicted to have lower average NPP (273, 543, and 1618 gC m^−2^ year^−1^ for algal turfs, and subtidal and intertidal seaweed forests, respectively; table S3), with the notable exception of areas with seasonal upwelling (e.g., Galápagos Islands, Oman). Seaweed productivity in most tropical reefs is dominated by algal turfs, which have low standing biomass but high rates of biomass turnover (*31*). The model predictions made here align with a recent global meta-analysis on algal turf productivity, which reports mean NPP rates <400 gC m^−2^ year^−1^ for most locations (*31*), reaffirming that seaweed NPP in the tropics falls well below that of temperate regions (table S3). Predicted NPP also decreased toward the poles, where some of the lowest NPP have been observed (e.g., <10 gC m^−2^ year^−1^ in Greenland and the Beaufort Sea). Seaweeds in polar regions face constant low temperatures and marked seasonality in light conditions driven by changing photoperiod and sea ice cover, which, at their polar limits, can limit growth to only 1 month per year and yield yearly net carbon balances close to zero (*32*). The decrease in NPP toward higher latitudes documented here agrees with previous studies across sub-Arctic to Arctic gradients (*33*), although data from polar regions were scarce (19 sites in the Arctic realm and 3 in the Southern Ocean).

### NPP patterns across primary producers

**Fig. 3.**
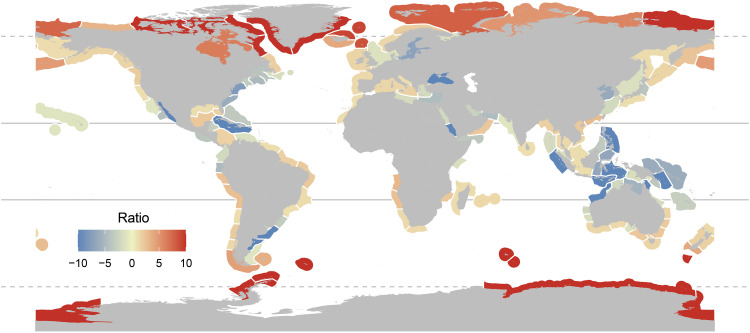
Relationship between subtidal seaweed forest and coastal phytoplankton productivity (gC m^−2^ year^−1^) across the world’s marine ecoregions. The ratio indicates the relative magnitude between subtidal seaweed forest and coastal phytoplankton per-area NPP, with positive ratios indicating seaweed forest NPP being greater. The ratio of ecoregions where NPP of marine forests or phytoplankton was not available is not shown. Lines indicate the tropics and polar circles.

**Fig. 4.**
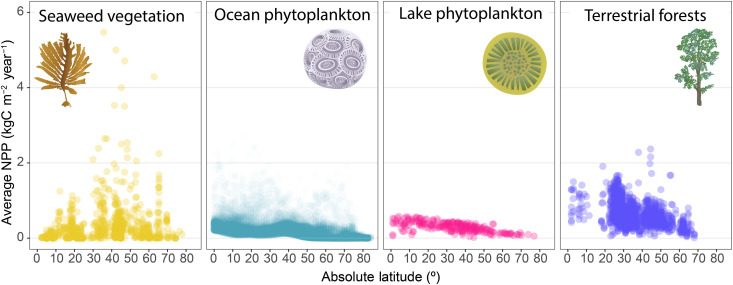
Relationship between latitude and NPP across Earth’s major primary producers. Lines depict the smoothed modeled mean. Terrestrial forest NPP includes above- and belowground productivity.

To identify coastal areas where seaweed productivity may be of most importance, we determined the relative magnitude of subtidal seaweed forest productivity—which are responsible for the bulk of seaweed carbon assimilation globally (*9*), to coastal phytoplankton, the other main carbon source to food webs in coastal areas (*1*, *6*, *15*). We found that the relative magnitude of seaweed forest carbon assimilation increases poleward, where per-area NPP can be more than 10 times greater than that of phytoplankton (Fig. 3). In contrast, phytoplankton dominated coastal productivity in multiple tropical locations and enclosed seas (e.g., Bohai, Baltic, Red, and Black Seas), where seaweed NPP is strongly limited by light, nutrients, temperature, and salinity (*34*). Seaweed NPP exhibited patterns opposite to those observed in terrestrial and freshwater primary producers, highlighting their spatially unique contribution to global carbon assimilation and its related biogeochemical cycles (Fig. 4).

## DISCUSSION

While seaweeds have long been known to be important contributors to coastal productivity (*13*), how that contribution varies spatially remains unresolved (*9*). Progress to date has been hampered by knowledge being based on observations made at small spatial scales and on a few well-studied taxa, mostly a few species of kelps [e.g., (*13*) and (*27*)]. Here, we overcome these limitations by (i) massively expanding the taxonomic and spatial coverage of NPP measurements [e.g., from the seminal kelp observations of (*13*) at three locations to >400 sites across the world], (ii) identifying the patterns and drivers of seaweed NPP at the global scale, and (iii) using this information to generate models predicting the productivity rates of different seaweed vegetation types across the coastal ocean.

Our observations suggest that the contribution of seaweeds to coastal productivity may be greater in temperate and polar environments. This has important consequences for coastal food webs, suggesting that seaweeds are key sources of carbon and energy in these environments, which aligns with stable carbon isotope studies reporting large contributions of seaweed-derived detritus to the diets and biomass of consumers (*35*, *36*). Our findings also suggest that the export of that productivity to the open ocean or deep sea may be greater toward high latitudes, which aligns with available reports of seaweeds on or in deep sea sediments (*11*). An important next step will be resolving the areal extent of seaweed habitats at high resolution, which will help determine the absolute magnitude of their carbon fluxes. The seaweed forest biome alone is estimated to cover more than 2 million km^2^ (*37*), likely playing a key role.

The strong climatic forcing on NPP identified in this study suggests that environmental change might have important effects on the global carbon assimilated by seaweed habitats, both through changes in metabolic rates and shifts in geographical distributions. In polar regions, warming, glacial retreat, and sea ice loss are driving increases in seaweed forest biomass, extent, and productivity (*38*, *39*). In contrast, warming is driving decreases in carbon assimilation and export in temperate regions experiencing seaweed forest loss and habitat reconfigurations (*40*), while mixed effects are expected on tropical seaweed habitats (*41*). Whether, at the global scale, NPP gains will offset losses remains to be determined, but it is clear that these changes will have important ecological consequences at regional scales. In addition, large-scale changes in environmental variables may interact with small-scale drivers such as turbidity, wave action, or epiphyte cover, yielding complex NPP responses at regional or local scales. For instance, the spatial intensity of “underwater Arctic greening” is variable, with increases in wind-driven sediment resuspension following ice breakup, increased riverine sediment delivery, and land runoff nullifying the benefits of increased irradiance and temperature in many locations (*42*).

Although our study provides the most robust estimates of global seaweed productivity rates to date, several sampling biases and shortcomings may have contributed error to our models. First, studies used to build the models did not randomly target the coastal vegetation of a region where productivity was modeled, but rather targeted specific groups and depths depending on the objective of the study. As a result, there was an uneven distribution of data across biogeographical regions, with fewer studies in the Southern Hemisphere (particularly southern Africa and South America), oceanic islands, and polar regions. Measuring seaweed NPP in climate envelopes that were not considered in this study should be a priority for future research to improve the accuracy of the predictions, particularly for groups where the data are globally scarce or spatially clustered (e.g., algal turfs). Second, 51% of studies included in our modeling effort considered only a single species or taxa (i.e., the focus of the study), and therefore, rates may underestimate the productivity of the entire seaweed assemblage (e.g., epiphytes or unaccounted understory algae in seaweed forests). Overall, however, ~80% of our dataset consisted of records of forest-forming algae and algal turfs, which are the predominant primary producers in coastal seaweed habitats (*9*, *15*, *20*, *31*). In addition, these seaweeds are responsible for the majority of biomass and NPP in their respective habitats, with understorey and epiphytes contributing minorly to total NPP in seaweed forests for example (*43*, *44*).

Third, our models were mostly informed by NPP measurements based on biomass accumulation methodologies, which typically do not account for losses due to grazing. Systematic differences in herbivory across space could therefore lead to consistent underestimates in areas with high grazing intensity. For instance, grazing intensity is generally believed to increase toward low latitudes. However, evidence for such patterns is mixed in marine systems (*45*, *46*), and tropical NPP was mostly derived from photorespirometry measurements or relied on biomass accumulation rates in herbivore-exclusion experiments, suggesting that unrecorded NPP loss to herbivory cannot explain differences between tropical and temperate latitudes. Fourth, studies varied in the time frame of their measurements (e.g., monthly, seasonally, and annually). This could lead to underestimates in regions where NPP is highly seasonal, such as temperate and polar seaweed forests (*47*), as well as underestimates from studies sampling at longer time frames, which does not account for grazing or detritus production in between measurements. However, sampling intensity showed no consistent trends with NPP in the taxa most widely sampled (fig. S8). In addition, NPP appears to vary little seasonally and interannually in tropical algal turfs (*48*, *49*), presumably reducing any artifact effects of sampling frequency and timing in this groups. Last, NPP studies on seaweed vegetation are typically short (1 to 2 years), with only 2% of studies conducting measurements ≥3 years and only three records conducting measurements >10 years. Hence, shorter-term studies may have failed to capture the long-term average NPP of a given site.

Overall, our work demonstrates that seaweeds, particularly seaweed forests, are important primary producers at a global scale and highlights the need to integrate them in oceanic carbon budgets, where they traditionally have been overlooked. In addition, as seaweeds form the largest coastal vegetated habitat globally (*5*), the maps and models presented provide a key resource to assess the regional and global contribution of marine vegetation to near-shore productivity, carbon fluxes, and food webs. An important next step will be resolving the fate of the carbon assimilated by seaweed habitats within different oceanographic settings and identifying which ocean processes and environmental variables drive these fluxes. These insights will allow a clearer view of the ecological processes, patterns of biodiversity, and biogeochemical fluxes taking place within the coastal ocean and will be important to predict how they may respond to the ongoing climatic change. Harnessing the productivity of seaweeds also offers promising opportunities to help meet the world’s future food security and contribute toward greater sustainability, with the work conducted here providing insights as to where greatest growth can be expected when scaling up seaweed aquaculture in coastal areas.

## MATERIALS AND METHODS

### Data compilation

#### Macroalgae NPP

We used a combination of published reports, peer-reviewed studies, and PhD theses to generate the most extensive dataset to date on seaweed NPP rates per area of seafloor.

The dataset, the references included in it, and a full description its characteristics and the methods used to compile it can be found in (*23*, *50*). Briefly, we first conducted a formal search of the literature in the Scopus database using the search terms “primary AND product* OR growth or npp and (seaweed OR alga* OR kelp OR rocky AND reef OR turf OR temperate AND reef OR coral OR polar OR Arctic),” which yielded 470 entries (September 2020). We initially filtered these results for potentially relevant studies based on the title and abstract, yielding 60 studies. Additional searches were performed in other literature repositories to capture studies conducted or published in non-English speaking countries (with English abstracts) (China National Knowledge Infrastructure database, China; J-STAGE repository, Japan; Scientific Electronic Library Online, South America). Additional studies were sourced from existing reviews on the productivity of tropical (*31*, *41*), temperate (*51*), and polar algae (*32*) and from being cited in the scanned papers. This yielded a total of 288 potentially relevant studies dealing with NPP of seaweed forests and other seaweed vegetation.

#### Terrestrial forest, freshwater, and oceanic phytoplankton NPP data

We used a range of NPP data from other primary producers to compare and contrast our findings. Terrestrial forest NPP data were derived from the Global Primary Production Data Initiative (*52*), which contains measurements of total NPP (i.e., above- and belowground NPP) obtained using a variety of different methods. The dataset covered 1921 sites dominated by forests from different biomes and was compiled from existing collections of data with no systematic standardization between studies [data defined as “class B” by Olson *et al*. (*52*)]—similar to the seaweed vegetation NPP database assembled here. Freshwater phytoplankton NPP data were extracted from (*53*), wherein authors quantified the NPP of satellite-observable lakes using a depth-integrated model that requires chlorophyll a and irradiance values and a light-utilization index (a latitude-dependent phytoplankton photosynthetic rate). Oceanic phytoplankton NPP data were extracted from the queryable layers provided as part of the Marine Socio-Environmental Covariates initiative (*54*). We selected the 2003–2013 annual averages of phytoplankton NPP, which are based on satellite measurements of PAR, Sea surface temperature, and chlorophyll and are produced by National Oceanic and Atmospheric Administration CoastWatch. The data are modeled on a 2.5–arc min grid and contains filtered cells >30 m depth. To compare the productivity of coastal phytoplankton with that of seaweed forests, we filtered cells <300 m in each ecoregion.

### Data selection and quality control

#### Macroalgae NPP

The list of potentially relevant NPP studies was then evaluated against a set of criteria to determine whether they provided reasonable estimates of a site’s areal carbon production at sufficient time resolution (i.e., gC m^−2^ year^−1^).

- First, studies had to examine NPP or biomass accumulation on a per-area basis. This criterion excluded studies examining biomass-specific productivity rates (i.e., gC g^−1^ m^−2^ year^−1^) unless those rates were applied to standing biomasses or covers in the field.

- Second, studies had to provide discrete estimates of NPP at the primary producer level (i.e., seaweed species or assemblage) with minimal interference of other photosynthetic or heterotrophic organisms. This criterion excluded studies examining net ecosystem primary production and metabolism when the NPP of the seaweed component could not be accurately determined, which usually rely on diel dissolved oxygen measurements in the water column.

- Third, studies had to capture seasonal variability in NPP across the year. This criterion excluded studies conducted at a single point in time, month or season, with the exception of studies concerning annual species where the growth or biomass accumulation was measured at the end of the life cycle (i.e., the maximum period of growth). The annual sampling frequency of each study (e.g., monthly, bimonthly, and seasonal) was noted for each study.

-Fourth, quantification of productivity had to be performed in situ on the reef or outdoor mesocosms mimicking natural reef conditions. This criterion excluded laboratory-only experiments, models (e.g., Ecopath models) and field studies in which the natural environmental conditions were experimentally modified (e.g., nutrient enrichment, acidification, and sediment additions).

- Fifth, details of the specific sampling location and measuring method had to be provided.

- Sixth, studies had to provide basic data not previously reported in other publications.

After applying the criteria above, our final filtered dataset featured 227 independent studies containing NPP data from 429 sites and 246 species or taxonomic entities (e.g., crustose coralline algae and algal turf). Entries were then classified according to the seaweed habitat where measurements were taken (Table 1). These habitats were defined on the basis of key structural parameters like vegetation height [e.g., seaweed forests sensu (*55*) versus seaweed beds, i.e., low-lying algal vegetation], the dominant vegetation (e.g., brown, red, or green algae), and their position within the water column (benthic or pelagic). Within a study, taxa from different groups could be classed in the same habitat (e.g., canopy, epiphytes, and understory algae all contributing to the NPP of a seaweed forest) unless they formed distinct patches within the habitat matrix (e.g., red algal bed patches interspersed with seaweed forests) or the study examined different depth bands, sites, or habitats.

Among the different seaweed vegetation types, subtidal seaweed forests were the most intensively studied vegetation (63.7% of the database records; fig. S1) followed by algal turfs (14.5%), red algal beds (8.8%), green algal beds (8.6%), intertidal red algal beds (8%), coralline algae and rhodolith beds (2%), brown algal beds (0.9%), other free floating algae (0.9%), and floating *Sargassum* (0.6%). When multiple and distinct species, spatial locations, depths, or methods were examined separately within a site or study, these were entered as separate case studies (separate rows). A wide range of metadata was also recorded for each study and can be found in (*23*). Briefly, this included information on the vegetation type, taxa examined, whether the study measured the NPP of single or multiple species, whether NPP required aggregation (e.g., for species in the same sampled depth and area, such as canopy and understory seaweeds), latitude and longitude, depth range, duration of the study, sampling frequency, and method used to obtain the data and method used to calculate NPP. In our study, a record was considered to be the per-area net primary productivity over the course of a year. If the data were not directly reported as annual rates, then these were computed on the basis of the monthly, bimonthly, or seasonal means (note that studies with low temporal sampling frequency were not included as per criteria above). Values reported in fresh or dry weight were converted to carbon using species-, genus-, family-, or order-specific factors when these were not available for a given species. Conversion factors provided in the studies were preferably used (e.g., photosynthetic quotient and carbon content), but, otherwise, these were derived from the database provided in (*56*).

### Modeling global net primary productivity per unit seafloor area

To predict the NPP of selected seaweed habitats across the world’s coastlines, we used a data-driven modeling approach comprising two steps. First, we parameterized NPP observations from a given site in relation to a range of environmental variables selected from known ecophysiological relationships with seaweed NPP (table S4) and one of the two methodologies used to measure carbon assimilation: biomass accumulation or photorespirometry. This resulted in two different predictions of NPP, which were then averaged to give a conservative estimate of the NPP at a given cell. We then used a set of high-resolution marine climatic data layers (*57*), and a fine-tuned dataset of the distribution of seaweeds, to apply the modeled relationships to the world’s coastlines. To account for areas where habitat may not be suitable for different vegetation types, we only modeled NPP where presence records of each vegetation type existed. The distribution of seaweed forests was derived from a dataset of 2.8 million records sourced from herbaria, the literature, and data repositories (*58*), with potential distribution gaps between records being filled based on the environmental niche where records occurred (*59*). Algal turfs were considered to be widespread across tropical and temperate latitudes (*31*), with predictions being limited between latitudes of observed algal turf records. The NPP of other vegetation could not be modeled due to their reduced number of estimates or absence of distribution estimates.

For our modeling efforts, we only included NPP estimates obtained via biomass accumulation or photorespirometry methods, as these are the most widespread and showed consistent patterns with latitude (fig. S1). This step excluded only three records (0.3%) from the total database. If a study provided the production of different species separately, but these were part of the same sampled area of seabed (e.g., multispecies *Sargassum* forest, canopy, and understory seaweeds), then the measurements were summed to yield the total NPP per area of seafloor. For 63 sites where NPP of seaweed vegetation was obtained across multiple depths, we selected the maximum NPP recorded at the study site for each of the studied vegetation types and by study year, study reference, and measuring method. This was done to achieve comparability with other locations as the majority of records came from shallow depths (<20 m), and downweighing the NPP of multidepth sites with low rates recorded at higher depths would have negatively affected their comparability. Applying these criteria yielded a total of 84 and 221 sites for intertidal and subtidal seaweed forests, respectively, and 107 for algal turfs (a total of 412 sites and 227 independent studies).

Modeling was performed with the machine learning algorithm boosted regression trees (BRT) by fitting NPP per seaweed group against environmental predictors with Gaussian distributions. We chose this ensemble algorithm as it handles nonlinear relationships and complex interactions between predictors while reducing overfitting by optimal hyperparameterization and forcing predictors to produce monotonic responses. The environmental predictors were selected on the basis of known ecophysiological relevance for seaweed vegetation NPP and accessed from BIO-ORACLE 2.1 (*57*), an open-source database containing marine data layers for ecological and bioclimatic modeling at resolution of 5 arc min (approximately 9 km at the equator). Photosynthetically active radiation (PAR) on the seabed was obtained from a 21-year continuous dataset at 5–arc min spatial resolution and 2–arc min bathymetry (*60*). PAR was not included as a predictor for the intertidal model. Maximum wave energy was produced to match the Bio-ORACLE 5–arc min resolution by applying the nearest neighbor algorithm based on the data available in (*61*). This predictor is provided in six classes, with 1 for calm enclosed seas and 6 for highest-energy oceanic coasts, long period swells, and storms. Because the methods used to obtain NPP estimates in the multiple studies compiled may strongly contribute to data variability, the “method” was also added as a predictor variable.

A 10-fold cross-validation framework was implemented to select the optimal BRT hyperparameters potentially reducing overfitting (*62*). In this process, all hyperparameter combinations (i.e., the “grid search” method), number of trees (50 to 1000, step 50), tree complexity (0.01 to 0.001, step 0.001), and learning rate (1 to 5) were tested in 10% of data withheld at a time with deviance explained (*62*). To further reduce overfitting, monotonic responses were forced to the predictor layers, except for the NPP method, according to the expected outcome in the response of models (*63*). Final predictions using optimal parameters were produced as maps at global scales as well as by aggregating NPP per ocean climatic zones and marine realms (*64*). Because the predictor method was not possible to extrapolate worldwide, predictions were performed by averaging the outcomes resulting from each single method. To restrict NPP estimates to regions where the seaweed groups are actually distributed, predictions were clipped with the distribution estimates available for seaweed forests or algal turfs (*58*). The final performance of the models was assessed using deviance explained against all NPP observations, and their significance was explored by determining the contribution of each predictor to the models (*62*).

## Supplementary Materials

### This PDF file includes:

Tables S1 to S4 Figs. S1 to S8 References
