# Supplementary material for: Global seaweed productivity
Source: Sci Adv. 2022 Sep 14;8(37):eabn2465. doi: 10.1126/sciadv.abn2465 (PMC9473579; doi:10.1126/sciadv.abn2465)
Supplement: Supplementary file 1 — Tables S1 to S4 Figs. S1 to S8 References [file sciadv.abn2465_sm.pdf]

Science Advances
AAAS

Supplementary Materials for

Global seaweed productivity

Albert Pessarrodona *et al.*

Corresponding author: Albert Pessarrodona, albert.pessarrodona@uwa.edu.au, pessa3@gmail.com

Sci. Adv. **8**, eabn2465 (2022)
DOI: 10.1126/sciadv.abn2465

This PDF file includes:

Tables S1 to S4
Figs. S1 to S8
References

Supplementary material

Table S1. Summary of the methods used to estimate NPP in seaweed forest their advantages and disadvantages. Biomass accumulation methods generally underestimate NPP as they only measure the carbon destined to tissue growth, whilst Photorespirometry. Free-water measurements integrate the metabolism of the whole benthic community, and therefore underestimate plant NPP as they also consider respiration by heterotrophs.

Method	Description	Spatial resolution	Temporal resolution	Advantages	Disadvantages
BIOMASS ACCUMULATION					
Periodic harvest of biomass	Periodic harvests of standing biomass over short time scales. Estimates are generally obtained at community level. Changes in standing biomass are attributed to growth or losses. Production can be estimated by subtracting the maximum and minimum biomass achieved, summing of all positive increments, or by counting individuals of a cohort and their mean weight through time (Allen method).	Community-scale	Weeks to Months	Widely available in the literature and easy to measure. Can cover spatially and temporally well.	Only measures assimilated carbon destined for growth and misses losses between sampling events (underestimate of NPP).<br>Does not measure individual growth rates<br>Not very suitable if species do not show a marked seasonality (e.g. in the tropics)
Increases in individual plant biomass	Individual plant increases in weight are followed through time by tagging, staining or punching holes in the plant. The mean individual increases in biomass are then multiplied by plant density. Can account for biomass losses when sampling frequently.	Individual- to community-scale	Weeks to Months	Widely available in the literature and easy to measure. Captures individual biomass accumulation/loss well	Only measures assimilated carbon destined for growth (underestimate of NPP).<br>Different parts of the thallus grow at different rates<br>Typically considers only adults, and a single species<br>Challenging for species with the meristem in the apical tips of the thallus
Photorespirometry incubations	Enclosing entire individuals (or communities) in transparent chambers to measure changes in DO (or more rarely CO_2). Measures true NPP (carbon assimilation) by subtracting respiration from gross primary productivity. Respiration is obtained by enclosing individuals in dark chambers. Estimates are obtained at individual and sometimes community level.	Individual- to community-scale	Hrs to days	Provide a more direct measure of photosynthesis and NPP.	Generally suitable for only small seaweeds<br>Oxygen methods requires PQ[1] (not always available)<br>Wide variation in the methods used by different authors.<br>Very susceptible to intrinsic sources of metabolic variation arising from thallus state (e.g. thallus age, part, reproductive state, morphology, crowding, macro and microhabitat, desiccation and injury)<br>Experimental setup used can significantly affect results: chamber volume, thallus weight, oxygen saturation levels, self-shading and water movement

					Limited in space and time, rarely considers daily, monthly and seasonal variation.<br>Typically considers only single or few species<br>Chamber can restrict water movement<br>Needs to consider background photosynthesis by phytoplankton
Incubations with carbon isotope tracers (^{13}C and ^{14}C).	Thalli are submerged in water enriched with isotopes and uptake by macroalgal tissue is measured after a given period of time. Measures true NPP.	Individual- to community-scale	Hrs to days	Directly estimates carbon assimilation	^{14}C methods generate expensive, hard to dispose radioactive seawater<br>Both methods only provide a measure of GPP.<br>Isotope labelling can be complicated *in situ.*<br>Not very common in the macroalgal literature
Free-water dissolved oxygen measurements (FOM) and Aquatic Eddy Covariance EAC)	Estimates community-scale GPP and respiration based on DO measurements through time and estimates of O_2 exchange with the atmosphere and vertical and horizontal advection.	Ecosystem-scale	Hrs to days	Measures whole community metabolism. Integrates production over large spatial areas	Reliant on PQ[1]<br>Assumptions associated with advection, quantification of the air-water exchange and respiration estimates required<br>Suitable only for simple habitats in sheltered environments<br>Limited to small space and time scales<br>However, given that typically only a few O_2 sensors can be deployed, FOM techniques miss important spatial heterogeneity<br>Includes respiration of heterotrophic components of the community
**ACOUSTICS**					
Underwater acoustics	The effect of gas bubbles produced during photosynthesis affects the propagation of sound. Changes on the acoustic signal can be correlated to primary production after some calibration.	Ecosystem-scale	Days	Measures gaseous oxygen produced. Integrates production over large areas	Acoustic properties of the bubbles vary with temperature and salinity.<br>Requires measures of species-specific acoustic properties.<br>Requires estimates of bathymetric profile<br>Rarely available in the literature.
**FLUOROMETRY**					
Pulse-amplitude-modulated (PAM) fluorometry	Chlorophyll *a* (Chl *a*) fluorescence measurements of PSII to obtain potential photosynthetic performance. Estimates a series of parameters that correlate with photosynthetic rates. Estimates are obtained at tissue level.	Tissue-scale	Minutes	Simple and convenient, non-invasive, fast	ETR only correlates well with photosynthetic rates at low irradiances and in thin thalli.<br>Indication of maximum potential photosynthesis only.<br>Measurements are focused on a small piece of tissue which may not be indicative of overall physiology of individual.<br>PSII activity need not equate to production of O_2.

[1]Photosynthetic Quotient, the molar ratio of oxygen released to the carbon dioxide assimilated during photosynthesis

Table S2. Maximum and mean net primary productivity (g C m^{-2} y^{-1}) of the major biomes on Earth and some agricultural crops.

Vegetation	Max	Mean ± SD	Reference
TERRESTRIAL			
Tundra	427	99 ± 87	(*51*, *64*)
Desert	902	216 ± 277	
Grassland	1680	353 ± 87	
Boreal forest	1550	449 ± 225	
Tropical forest	2013	765 ± 490	
Subtropical forest	1756	773 ± 302	
Temperate forest	1755	813 ± 331	
Wetland	3920	886 ± 754	
Savanna	1700	916 ± 567	
CROPS			
All crops		344	(*65*)
Small grains		294	
Coarse grains		309	
Sugar crops		801	
Coarse grains		309	
Pulses		181	
Vegetables		218	
OCEAN			
Seaweed			
Algal turfs	829	344±111	This study
Subtidal seaweed forests	4768	655±741	
Intertidal seaweed forests	4417	1711±1140	
Phytoplankton			
Oceanic Phytoplankton	4562	149 ± 1.1	(*66*)
Estuarine Phytoplankton	1890	238 ± ND	(*67*)

Table S3. Mean and maximum modelled productivity (g C m^{-2} y^{-1}) across major ocean climatic zones and biogeographical realms. Parenthesis indicate the 95% confidence interval around the mean.

	Subtidal marine forests		Intertidal marine forests		Algal turfs	
**CLIMATE ZONE**	**Mean**	**Max**	**Mean**	**Max**	**Mean**	**Max**
Polar regions (<10°C)	986 (780-1193)	2606	2702 (2324-3080)	4141	385 (314-455)	532
Cold Temperate (10-15°C)	712 (532-892)	2489	2521 (2166-2876)	4147	391 (321-461)	550
Warm temperate (15-25°C)	582 (392-773)	4768	2561 (2197-2926)	4147	363 (296-431)	642
Tropical (>25°C)	543 (296-791)	4299	1618 (1373-1864)	4418	273 (207-339)	819
BIOGEOGRAPHIC REALM						
Southern Ocean	1758 (1423-2094)	2606	978 (811-1144)	2310	423 (344-502)	471
Temperate Southern Africa	1582 (1277-1887)	4768	777 (633-920)	1675	470 (394-546)	550
Temperate South America	1108 (835-1381)	4467	1683 (1428-1938)	2948	340 (271-408)	523
Temperate Australasia	804 (620-988)	4293	1062 (873-1251)	2849	443 (371-514)	642
Arctic	802 (615-988)	2588	2248 (1925-2571)	4418	381 (312-450)	532
Western Indo-Pacific	775 (502-1049)	4183	1200 (1003-1398)	3370	312 (240-384)	658
Tropical Atlantic	748 (476-1019)	4165	1698 (1458-1938)	3868	404 (331-477)	819
Tropical Eastern Pacific	703 (380-1025)	4266	1147 (966-1328)	2735	386 (301-471)	568
Temperate Northern Pacific	557 (368-746)	4101	1820 (1550-2091)	4262	312 (242-382)	532
Temperate Northern Atlantic	467 (285-649)	4301	1728 (1464-1991)	4303	300 (237-362)	582
Central Indo-Pacific	418 (145-692)	3574	1217 (1028-1407)	3837	247 (182-312)	548
Eastern Indo-Pacific	165 (4-326)	479	596 (449-744)	1919	641 (545-738)	657
**GLOBAL**	655 (0-2098)	4768	1711 (402-4187)	4418	344 (162-510)	829

Table S4. Summary of the environmental predictor variables used in our models and their documented relationships with NPP. All data was obtained from the BIO-ORACLE repository (*56*) except for PAR (*59*) and maximum wave energy (*60*).

Predictor	Limiting in	Examples	Resolution
Max. Temp. (°C)	Tropical enclosed seas (e.g., Persian Gulf, Red Sea), South China Sea	(*20*, *68*)	2.5 arcminute
Min. Temp. (°C)	Polar seas	(*32*)	2.5 arcminute
Nitrate[1] (μM)	Oligotrophic seas (e.g., Mediterranean, Caribbean Sea), tropical Pacific, some temperate regions (e.g., Nova Scotia)	(*20*, *69*, *70*)	15 arcminute
PAR (mol photons m^{-2} day^{-1})	Baltic Sea, Polar seas	(*32–34*, *70*)	5 arcminute
Salinity (PSU)	Estuarine areas, Baltic Sea	(*34*)	15 arcminute
Maximum Wave Energy (kWm)	Enclosed seas, sheltered areas	(*29*)	5 arcminute

[1]Nitrate was included as a proxy for nutrients. Phosphate was not included given that is highly correlation to Nitrate

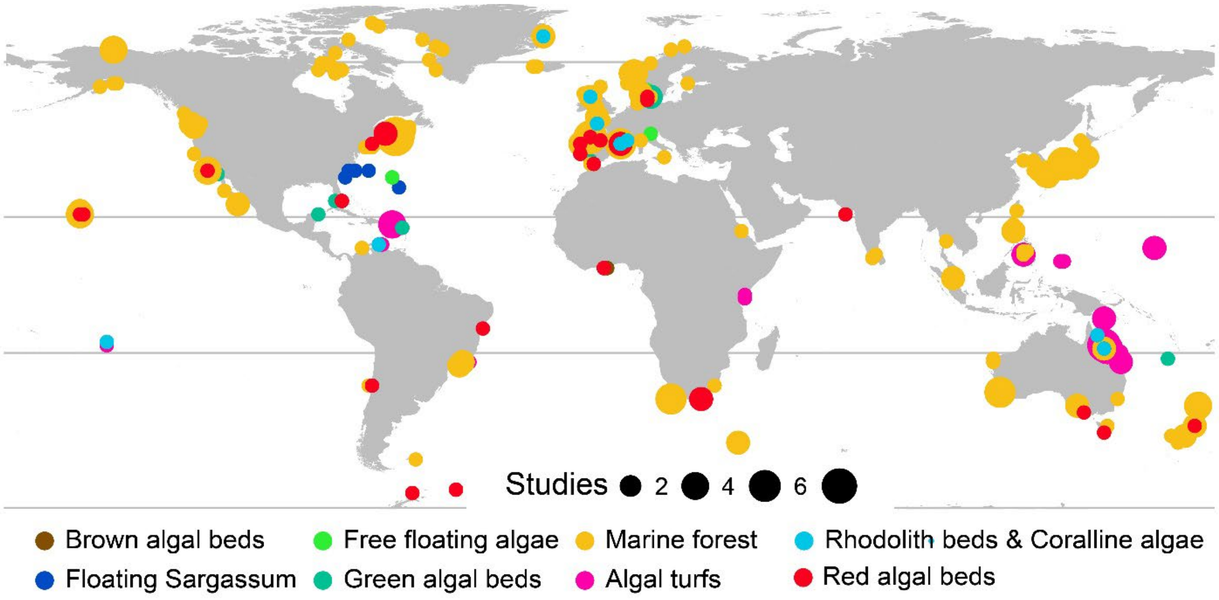

Figure S1. Location of the study sites included in the database. The size of the bubbles denotes the number of studies conducted in that location, while the colours denote the vegetation type studied.

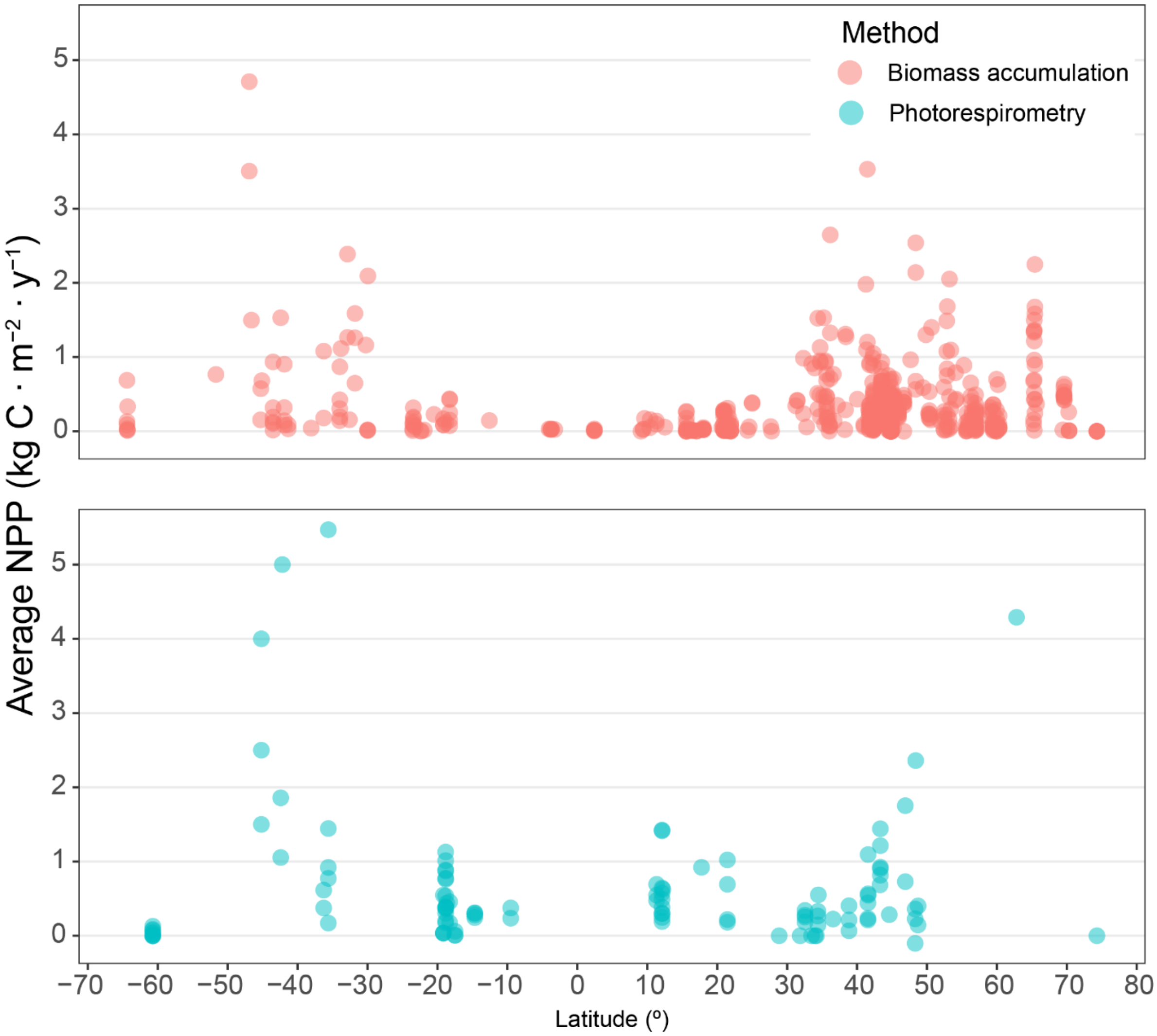

Figure S2. Latitudinal patterns of observed marine forest NPP depending on measuring methods. Dots indicate the average NPP of a study conducted within a given location.

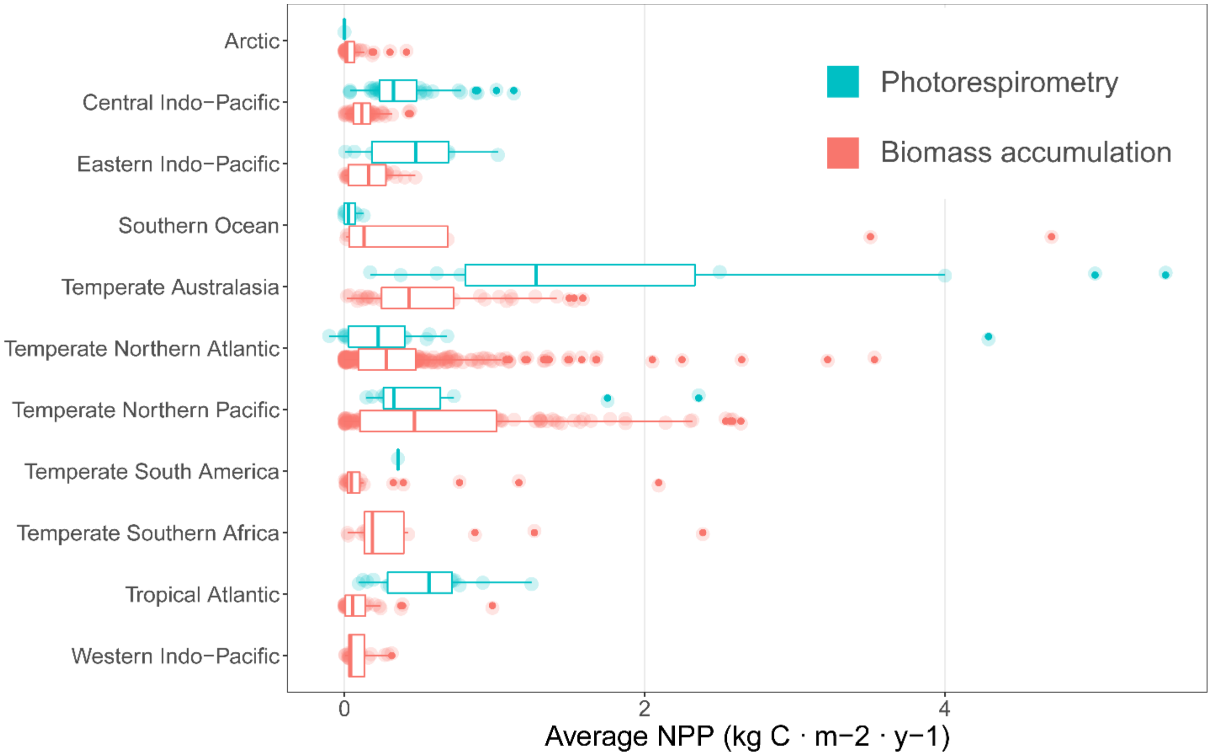

Figure S3. Average observed NPP by broad measuring methodologies across the major ocean biogeographic realms.

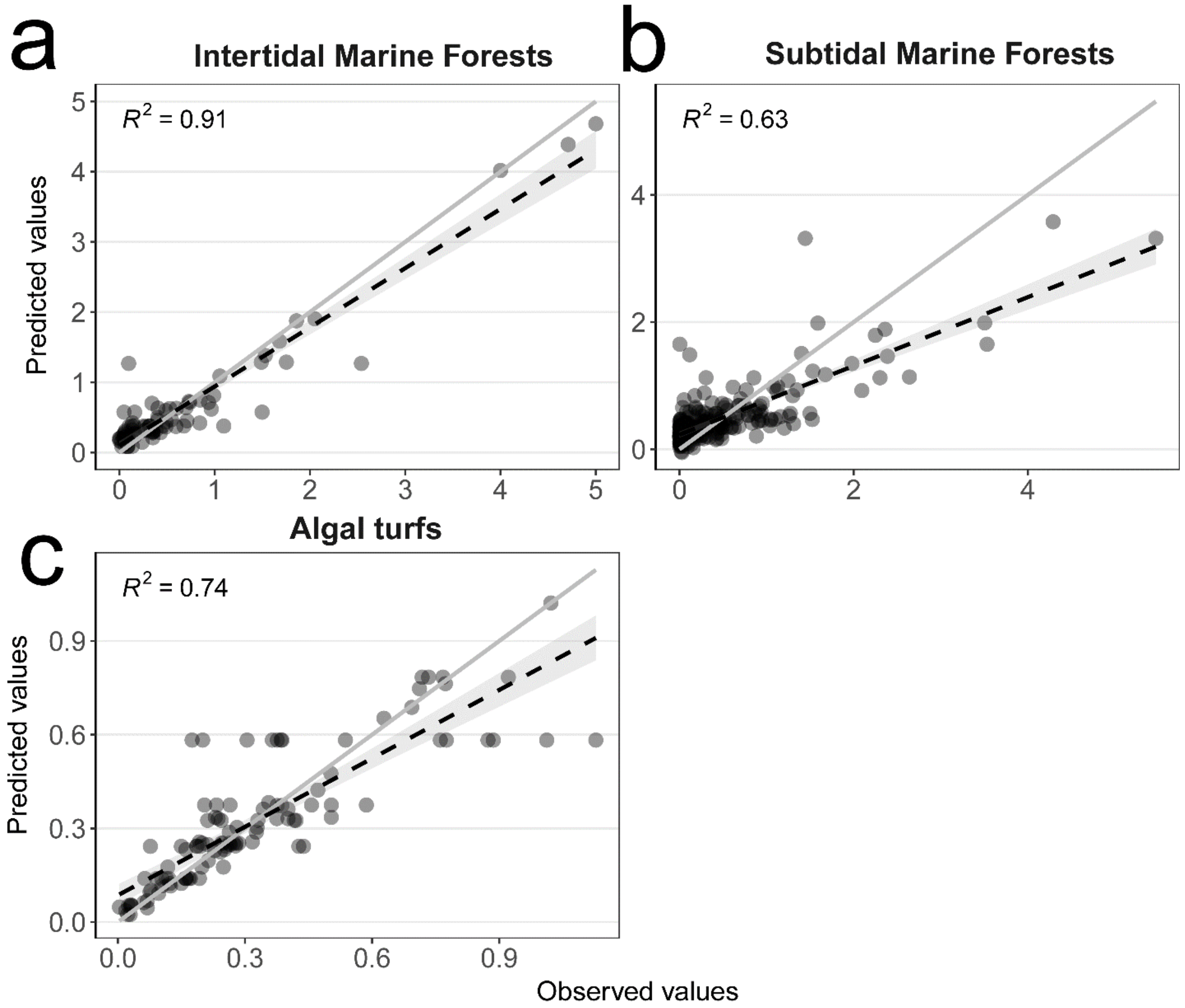

Figure S4. Model fits for (a) intertidal and (b) subtidal marine forests, and (c) other seaweed vegetation. Units are kg C m^{-2} y^{-1}. The grey straight line depicts the 1:1 fit, while the black dashed line and shaded grey area indicates the predicted relationship and standard error.

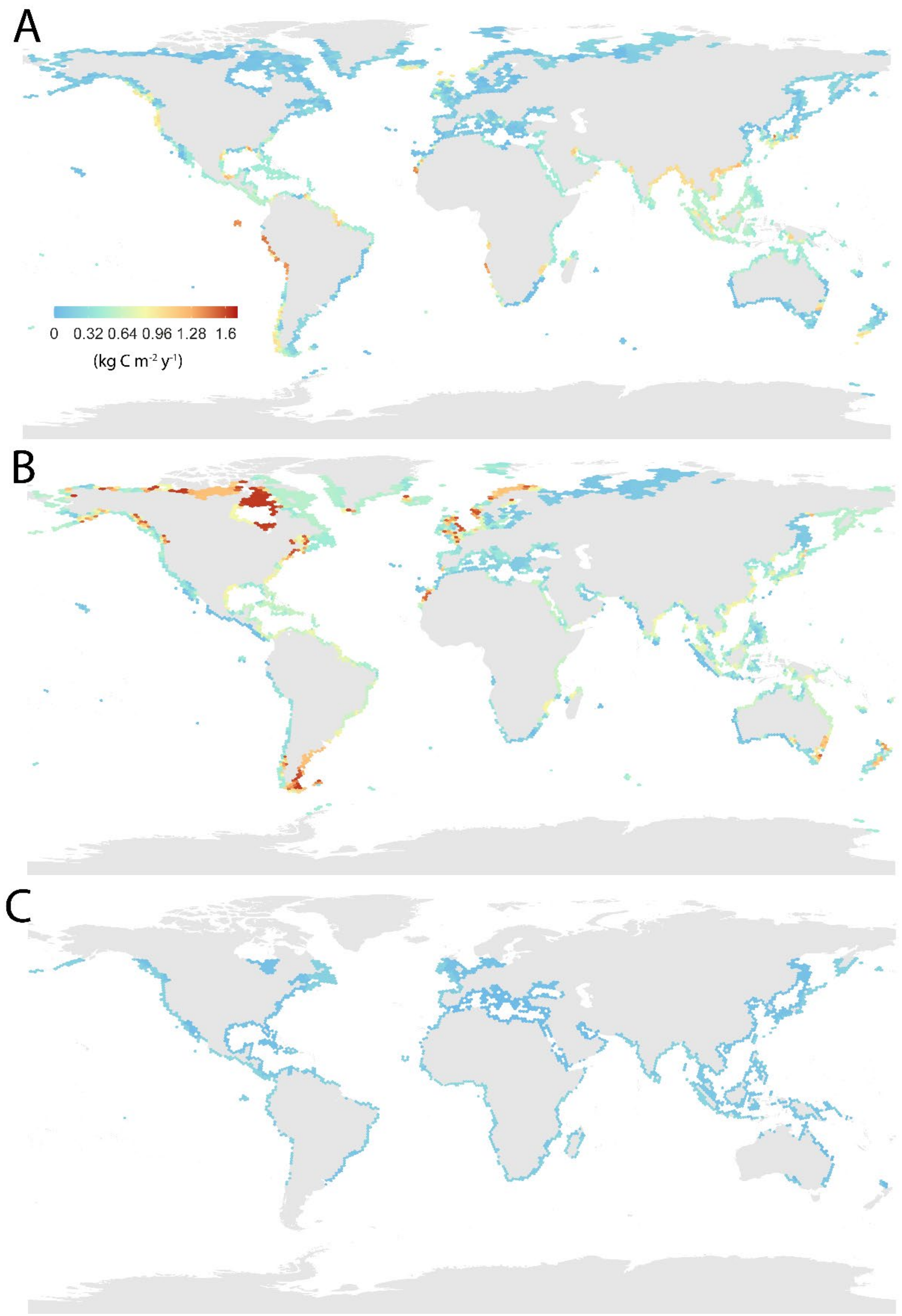

Figure S5. Standard deviation around the average model prediction (Fig. 1), obtained by averaging predictions made based on the biomass accumulation and photo respirometry methods for subtidal (a) and intertidal (b) seaweed forests and (c) subtidal algal turfs.

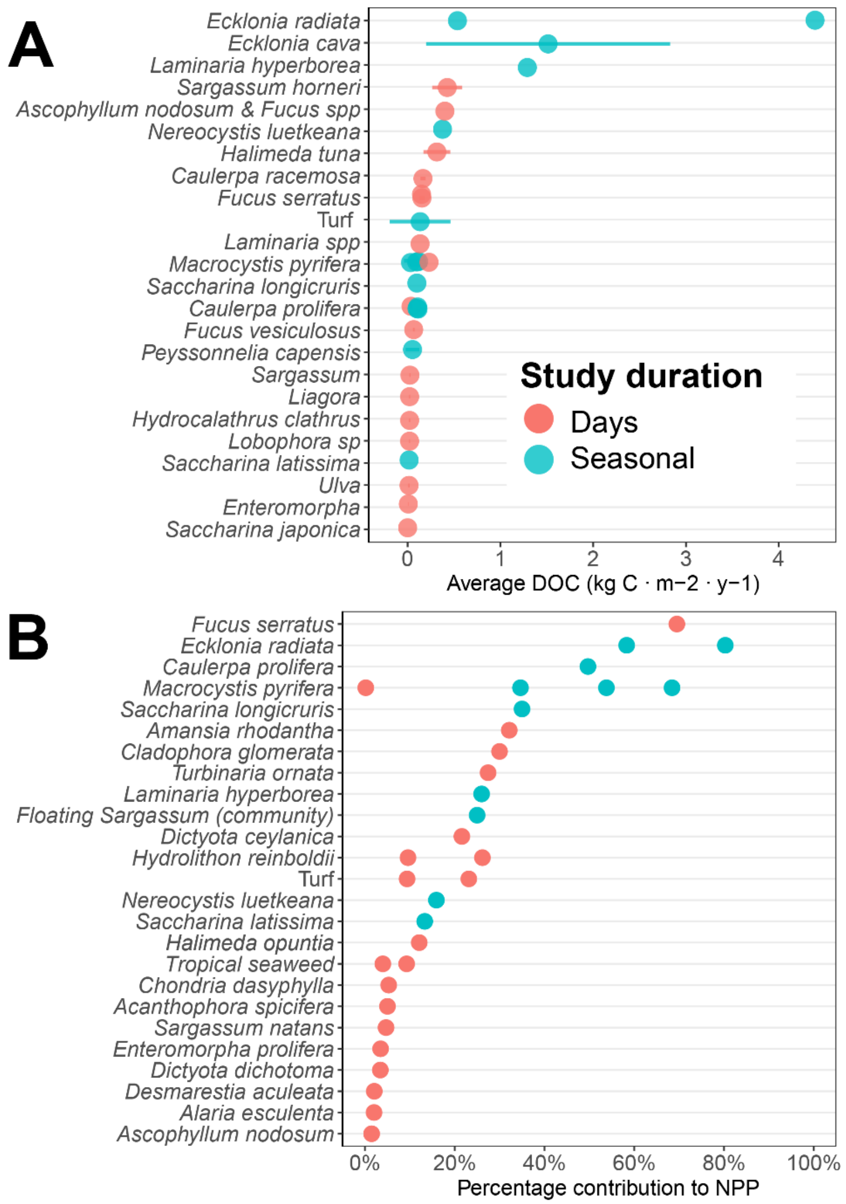

Figure S6. Species-level variability in DOC release rates (a) and its percentage contribution to total NPP (b). Dots and lines indicate the mean and standard deviation reported in a given study. Colours denote the timeframe over which the DOC release rate experiments (red: days to a week, blue: months to a year). Data was compiled from 26 different studies.

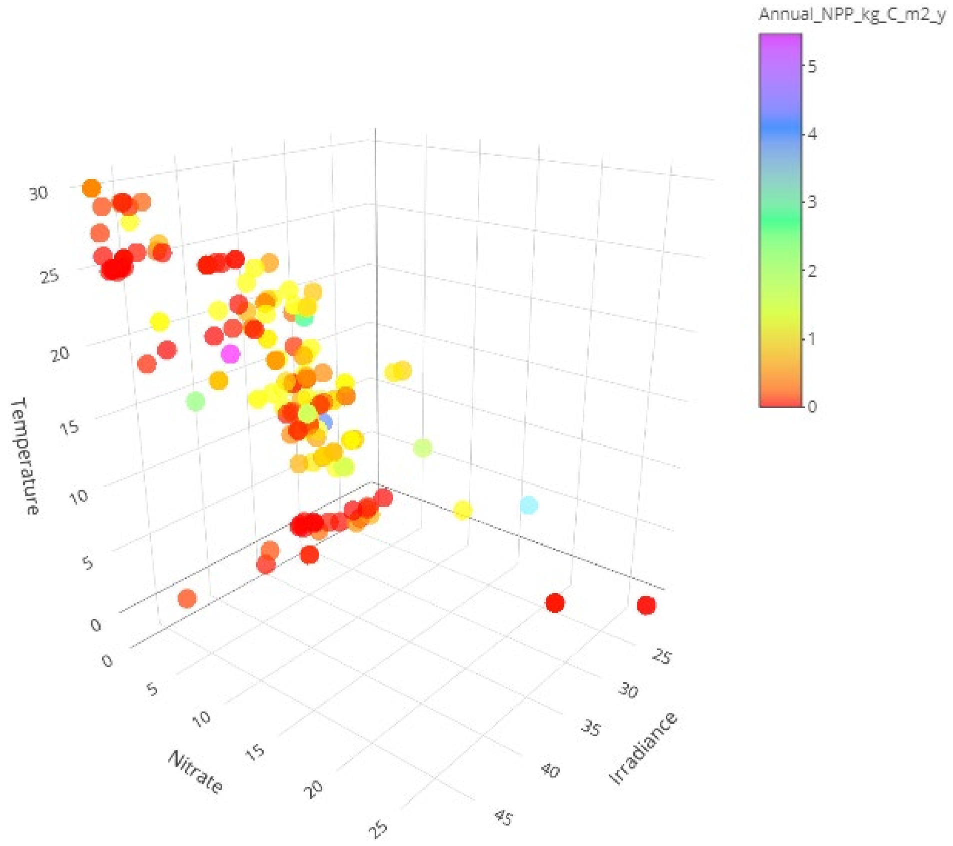

Figure S7. Observed NPP of seaweed forests in the climate space, represented by mean annual sea surface temperature (°C), irradiance (mol photon m^{-2} day^{-1}), and nutrient (nitrate) concentrations (>1 μM). Each data point represents an observation at one of the studied sites.

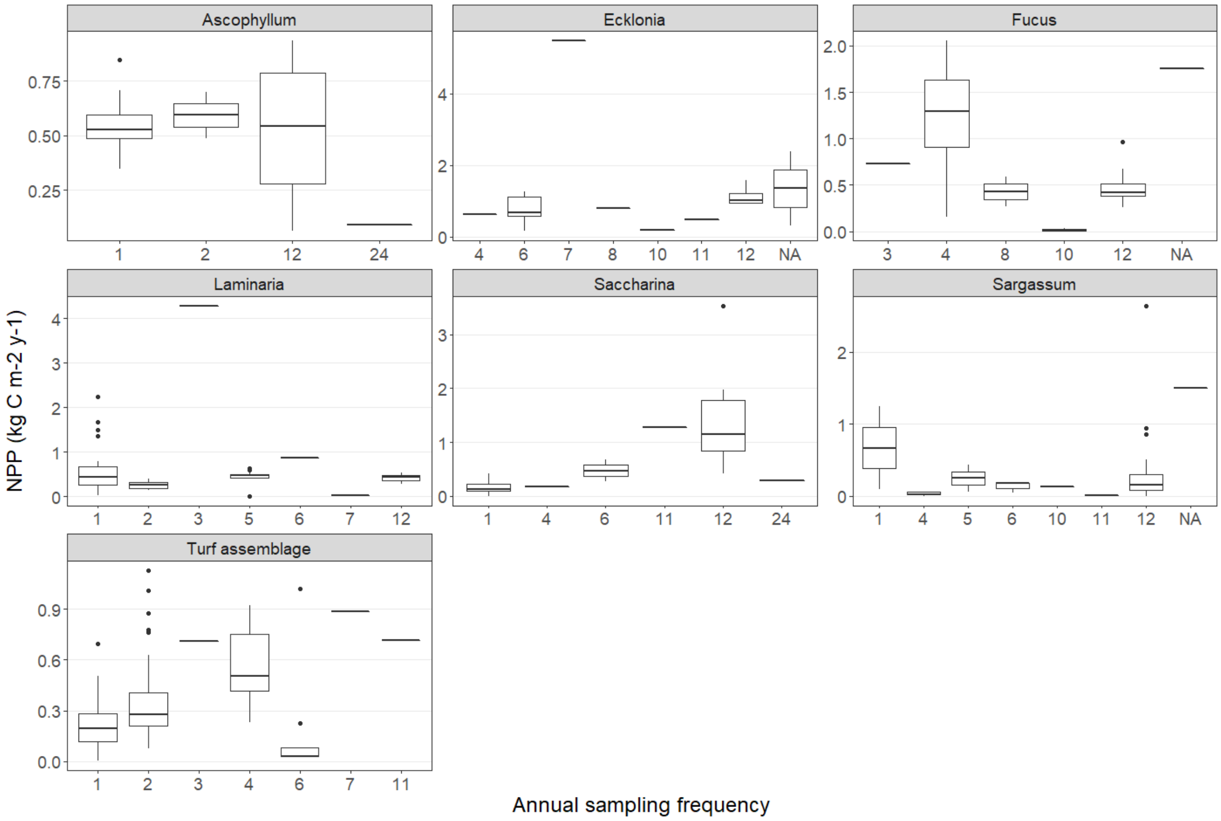

Figure S8. Boxplots showing the relationship between sampling intensity and NPP amongst the most intensively studied taxa.

REFERENCES AND NOTES

1. C. B. Field, M. J. Behrenfeld, J. T. Randerson, P. Falkowski, Primary production of the biosphere: Integrating terrestrial and oceanic components. *Science* **281**, 237–240 (1998).

2. M. J. Behrenfeld, R. T. O'Malley, D. A. Siegel, C. R. McClain, J. L. Sarmiento, G. C. Feldman, A. J. Milligan, P. G. Falkowski, R. M. Letelier, E. S. Boss, Climate-driven trends in contemporary ocean productivity. *Nature* **444**, 752–755 (2006).

3. H. M. Dierssen, R. C. Zimmerman, L. A. Drake, D. Burdige, Benthic ecology from space: Optics and net primary production in seagrass and benthic algae across the Great Bahama Bank. *Mar. Ecol. Prog. Ser.* **411**, 1–15 (2010).

4. V. S. Saba, M. A. M. Friedrichs, D. Antoine, R. A. Armstrong, I. Asanuma, M. J. Behrenfeld, A. M. Ciotti, M. Dowell, N. Hoepffner, K. J. W. Hyde, J. Ishizaka, T. Kameda, J. Marra, F. Mélin, A. Morel, J. O'Reilly, M. Scardi, W. O. Smith Jr., T. J. Smyth, S. Tang, J. Uitz, K. Waters, T. K. Westberry, An evaluation of ocean color model estimates of marine primary productivity in coastal and pelagic regions across the globe. *Biogeosciences* **8**, 489–503 (2011).

5. C. M. Duarte, I. J. Losada, I. E. Hendriks, I. Mazarrasa, N. Marbà, The role of coastal plant communities for climate change mitigation and adaptation. *Nat. Clim. Chang.* **3**, 961–968 (2013).

6. D. Krause-Jensen, S. Markager, T. Dalsgaard, D. K. Stiig, M. Tage, Benthic and pelagic primary production in different nutrient regimes. *Estuaries and Coasts.* **35**, 527–545 (2012).

7. J. P. Gattuso, B. Gentili, C. M. Duarte, J. A. Kleypas, J. J. Middelburg, D. Antoine, Light availability in the coastal ocean: Impact on the distribution of benthic photosynthetic organisms and their contribution to primary production. *Biogeosciences* **3**, 489–513 (2006).

8. J. Holt, J. Harle, R. Proctor, S. Michel, M. Ashworth, C. Batstone, I. Allen, R. Holmes, T. Smyth, K. Haines, D. Bretherton, G. Smith, Modelling the global coastal ocean. *Phil. Trans. R. Soc. A.* **367**, 939–951 (2009).

9. C. M. Duarte, J.-P. Gattuso, K. Hancke, H. Gundersen, K. Filbee-Dexter, M. F. Pedersen, J. J. Middelburg, M. T. Burrows, K. A. Krumhansl, T. Wernberg, P. Moore, A. Pessarrodona, S. B. Øberg, I. Sousa-Pinto, J. Assis, A. M. Queirós, D. A. Smale, T. Bekkby, E. A. Serrão, D. Krause-Jensen, Global estimates of the extent and production of macroalgal forests. *Glob. Ecol. Biogeogr.* **31**, 1422–1439 (2022).

10. A. Ortega, N. R. Geraldi, I. Alam, A. A. Kamau, S. G. Acinas, R. Logares, J. M. Gasol, R. Massana, D. Krause-Jensen, C. M. Duarte, Important contribution of macroalgae to oceanic carbon sequestration. *Nat. Geosci.* **12**, 748–754 (2019).

11. D. Krause-Jensen, C. M. Duarte, Substantial role of macroalgae in marine carbon sequestration. *Nat. Geosci.* **9**, 737–742 (2016).

12. L. R. Blinks, Photosynthesis and productivity of littoral marine algae. *J. Mar. Res.* **14**, 363–373 (1955).

13. K. H. Mann, Seaweeds: Their productivity and strategy for growth. *Science* **182**, 975–981 (1973).

14. S. V. Smith, Marine macrophytes as a global carbon sink. *Science* **211**, 838–840 (1981).

15. C. Charpy-Roubaud, A. Sournia, The comparative estimation of phytoplanktonic, microphytobenthic and macrophytobenthic primary production in the oceans. *Mar. Microb. Food Webs.* **4**, 31–57 (1990).

16. C. G. N. De Vooys, in *The Global Carbon Cycle. Scientific Committee on Problems of the Environment (SCOPE) of the International Council of Scientific Unions (ICSU)*, B. Bolin, E. T. Degens, S. Kempe, P. Ketner, Eds. (Wiley, 1979), pp. 259–292.

17. R. H. Whittaker, G. E. Likens, Carbon in the biota. *Brookhaven Symp. Biol.* **30**, 281–302 (1973).

18. B. H. Brinkhuis, Comparisons of salt-marsh fucoid production estimated from three different indices. *J. Phycol.* **13**, 328–335 (1977).

19. D. A. Smale, A. Pessarrodona, N. King, M. T. Burrows, A. Yunnie, T. Vance, P. Moore, Environmental factors influencing primary productivity of the forest - forming kelp *Laminaria hyperborea* in the northeast Atlantic. *Sci. Rep.* **10**, 12161 (2020).

20. K. Lüning, in *Seaweeds: Their Environment, Biogeography, and Ecophysiology* (John Wiley & Sons Inc., 1990).

21. A. L. Middelboe, K. Sand-Jensen, T. Binzer, Highly predictable photosynthetic production in natural macroalgal communities from incoming and absorbed light. *Oecologia* **150**, 464–476 (2006).

22. A. Anav, P. Friedlingstein, C. Beer, P. Ciais, A. Harper, C. Jones, G. Murray-Tortarolo, D. Papale, N. C. Parazoo, P. Peylin, S. Piao, S. Sitch, N. Viovy, A. Wiltshire, M. Zhao, Spatiotemporal patterns of terrestrial gross primary production: A review. *Rev. Geophys.* **53**, 785–818 (2015).

23. A. Pessarrodona, K. Filbee-Dexter, K. A. Krumhansl, P. J. Moore, T. Wernberg, A global dataset of seaweed net primary productivity. *Sci. Data* **9**, 484 (2022).

24. A. C. Cheshire, N. D. Hallam, Biomass and density of native stands of *DurvilIaea potatorum* (southern bull-kelp) in south eastern Australia. *Mar Ecol Prog Ser.* **48**, 277–283 (1988).

25. M. M. Littler, K. E. Arnold, Primary productivity of marine macroalgal functional-form groups from southwestern North America. *J. Phycol.* **18**, 307–311 (1982).

26. A. Migné, G. Delebecq, D. Davoult, N. Spilmont, D. Menu, F. Gévaert, Photosynthetic activity and productivity of intertidal macroalgae: In situ measurements, from thallus to community scale. *Aquat. Bot.* **123**, 6–12 (2015).

27. V. A. Fairhead, thesis, The University of Adelaide (2001).

28. D. A. Smale, M. T. Burrows, A. J. Evans, N. King, M. D. J. Sayer, A. L. E. Yunnie, P. J. Moore, Linking environmental variables with regional-scale variability in ecological structure and standing stock of carbon within kelp forests in the United Kingdom. *Mar. Ecol. Prog. Ser.*

542, 79–95 (2016).

29. M. F. Pedersen, L. B. Nejrup, S. Fredriksen, H. Christie, K. M. Norderhaug, Effects of wave exposure on population structure, demography, biomass and productivity of the kelp *Laminaria hyperborea*. *Mar. Ecol. Prog. Ser.* **451**, 45–60 (2012).

30. B. Konar, K. Iken, J. J. Cruz-Mota, L. Benedetti-Cecchi, A. Knowlton, G. Pohle, P. Miloslavich, M. Edwards, T. Trott, E. Kimani, R. Riosmena-Rodriguez, M. Wong, S. Jenkins, A. Silva, I. S. Pinto, Y. Shirayama, Current patterns of macroalgal diversity and biomass in northern hemisphere rocky shores. *PLOS ONE* **5**, e13195 (2010).

31. S. B. Tebbett, D. R. Bellwood, Algal turf productivity on coral reefs: A meta-analysis. *Mar. Environ. Res.* **168**, 105311 (2021).

32. I. Gómez, A. Wulff, M. Y. Roleda, P. Huovinen, U. Karsten, M. L. Quartino, K. H. Dunton, C. Wiencke, Light and temperature demands of marine benthic microalgae and seaweeds in polar regions. *Bot. Mar.* **52**, 593–608 (2009).

33. D. Krause-Jensen, N. Marbà, B. Olesen, M. K. Sejr, P. B. Christensen, J. Rodrigues, P. E. Renaud, T. J. S. Balsby, S. Rysgaard, Seasonal sea ice cover as principal driver of spatial and temporal variation in depth extension and annual production of kelp in Greenland. *Glob. Chang. Biol.* **18**, 2981–2994 (2012).

34. M. Pärnoja, J. Kotta, H. Orav-Kotta, T. Paalme, Comparisons of individual and community photosynthetic production indicate light limitation in the shallow water macroalgal communities of the Northern Baltic Sea. *Mar. Ecol.* **35**, 19–27 (2014).

35. K. H. Dunton, D. M. Schell, Dependence of consumers on macroalgal (*Laminaria solidungula*) carbon in an arctic kelp community: $\delta^{13}C$ evidence. *Mar. Biol.* **93**, 615–625 (1987).

36. P. E. Renaud, T. S. Løkken, L. L. Jørgensen, J. Berge, Macroalgal detritus and food-web subsidies along an Arctic fjord. *Front. Mar. Sci.* **2**, 00031 (2015).

37. D. R. M. Jayathilake, M. J. Costello, Version 2 of the world map of laminarian kelp benefits

from more Arctic data and makes it the largest marine biome. *Biol. Conserv.* **257**, 109099 (2021).

38. D. Krause-Jensen, P. Archambault, J. Assis, I. Bartsch, K. Bischof, K. Filbee-Dexter, K. H. Dunton, O. Maximova, S. B. Ragnarsdóttir, M. K. Sejr, U. Simakova, V. Spiridonov, S. Wegeberg, M. H. S. Winding, C. M. Duarte, Imprint of climate change on pan-Arctic marine vegetation. *Front. Mar. Sci.* **7**, 617324 (2020).

39. M. L. Quartino, D. Deregibus, G. L. Campana, G. E. J. Latorre, F. R. Momo, Evidence of macroalgal colonization on newly ice-free areas following glacial retreat in Potter Cove (South Shetland Islands), Antarctica. *PLOS ONE* **8**, e58223 (2013).

40. A. Pessarrodona, P. J. Moore, M. D. J. Sayer, D. A. Smale, Carbon assimilation and transfer through kelp forests in the NE Atlantic is diminished under a warmer ocean climate. *Glob. Chang. Biol.* **24**, 4386–4398 (2018).

41. C. J. Fulton, R. A. Abesamis, C. Berkström, M. Depczynski, N. A. J. Graham, T. H. Holmes, M. Kulbicki, M. M. Noble, B. T. Radford, S. Tano, P. Tinkler, T. Wernberg, S. K. Wilson, Form and function of tropical macroalgal reefs in the Anthropocene. *Funct. Ecol.* **33**, 989–999 (2019).

42. C. Bonsell, K. H. Dunton, Long-term patterns of benthic irradiance and kelp production in the central Beaufort sea reveal implications of warming for Arctic inner shelves. *Prog. Oceanogr.* **162**, 160–170 (2018).

43. R. J. Miller, D. C. Reed, M. A. Brzezinski, Partitioning of primary production among giant kelp (Macrocystis pyrifera), understory macroalgae, and phytoplankton on a temperate reef. *Limnol. Oceanogr.* **56**, 119–132 (2011).

44. L. W. Tait, D. R. Schiel, Dynamics of productivity in naturally structured macroalgal assemblages: Importance of canopy structure on light-use efficiency. *Mar. Ecol. Prog. Ser.* **421**, 97–107 (2011).

45. A. G. B. Poore, A. H. Campbell, R. A. Coleman, G. J. Edgar, V. Jormalainen, P. L. Reynolds, E. E. Sotka, J. J. Stachowicz, R. B. Taylor, M. A. Vanderklift, J. Emmett Duffy,

Global patterns in the impact of marine herbivores on benthic primary producers. *Ecol. Lett.* **15**, 912–922 (2012).

46. G. O. Longo, M. E. Hay, C. E. L. Ferreira, S. R. Floeter, Trophic interactions across 61 degrees of latitude in the Western Atlantic. *Glob. Ecol. Biogeogr.* **28**, 107–117 (2019).

47. A. Rassweiler, D. C. Reed, S. L. Harrer, J. C. Nelson, Improved estimates of net primary production, growth, and standing crop of *Macrosystis pryifera* in Southern California. *Ecology* **99**, 2132 (2018).

48. R. C. Carpenter, Mass mortality of *Diadema antillarum*: I. Long-term effects on sea urchin population-dynamics and coral reef algal communities. *Mar. Biol.* **104**, 67–77 (1990).

49. D. W. Klumpp, A. D. McKinnon, Community structure, biomass and productivity of epilithic algal communities on the Great Barrier Reef: Dynamics at different spatial scales. *Mar. Ecol. Prog. Ser.* **86**, 77–89 (1992).

50. A. Pessarrodona, J. Assis, K. Filbee-Dexter, M. T. Burrows, J.-P. Gattuso, C. M. Duarte, D. Krause-Jensen, P. J. Moore, D. A. Smale, T. Wernberg, Global seaweed productivity. https://doi.org/10.1038/s41597-022-01554-5 (2022).

51. T. Wernberg, K. Krumhansl, K. Filbee-Dexter, M. F. Pedersen, Status and trends for the world’s kelp forests, in *World Seas: An Environmental Evaluation: Ecological Issues and Environmental Impacts*, C. Sheppard, Ed. (Academic Press, 2019); https://linkinghub.elsevier.com/retrieve/pii/B9780128050521000036, pp. 57–78.

52. R. J. Olson, J. M. O. Scurlock, S. D. Prince, D. L. Zheng, K. R. Johnson, in *NPP Multi-Biome: Global Primary Production Data Initiative Products, R2* (Oak Ridge, 2013); https://doi.org/10.3334/ORNLDAAC/617.

53. M. J. Sayers, G. L. Fahnenstiel, R. A. Shuchman, K. R. Bosse, A new method to estimate global freshwater phytoplankton carbon fixation using satellite remote sensing: Initial results. *Int. J. Remote Sens.* **42**, 3708–3730 (2021).

54. L. A. Yeager, P. Marchand, D. A. Gill, J. K. Baum, J. M. McPherson, Marine socio-environmental covariates: Queryable global layers of environmental and anthropogenic variables for marine ecosystem studies. *Ecology* **98**, 1976 (2017).

55. T. Wernberg, K. Filbee-Dexter, Missing the marine forest for the trees. *Mar. Ecol. Prog. Ser.* **612**, 209–215 (2019).

56. T. Brey, C. Müller-Wiegmann, Z. M. C. Zittier, W. Hagen, Body composition in aquatic organisms—A global data bank of relationships between mass, elemental composition and energy content. *J. Sea Res.* **64**, 334–340 (2010).

57. J. Assis, L. Tyberghein, S. Bosch, H. Verbruggen, E. A. Serrão, O. De Clerck, Bio-ORACLE v2.0: Extending marine data layers for bioclimatic modelling. *Glob. Ecol. Biogeogr.* **27**, 277–284 (2018).

58. J. Assis, E. Fragkopoulou, D. Frade, J. Neiva, A. Oliveira, D. Abecasis, S. Faugeron, E. A. Serrão, A fine-tuned global distribution dataset of marine forests. *Sci. Data.* **7**, 119 (2020).

59. E. Fragkopoulou, E. A. Serrão, O. De Clerck, M. J. Costello, M. B. Araújo, C. M. Duarte, D. Krause-Jensen, J. Assis, Global biodiversity patterns of marine forests of brown macroalgae. **31**, 636–648 (2022).

60. J.-P. Gattuso, B. Gentili, D. Antoine, D. Doxaran, Global distribution of photosynthetically available radiation on the seafloor. *Earth Syst. Sci. Data* **12**, 1697–1709 (2020).

61. I. Fairley, M. Lewis, B. Robertson, M. Hemer, I. Masters, J. Horrillo-Caraballo, H. Karunarathna, D. E. Reeve, A classification system for global wave energy resources based on multivariate clustering. *Appl. Energy* **262**, 114515 (2020).

62. J. Elith, J. R. Leathwick, T. Hastie, A working guide to boosted regression trees. *J. Anim. Ecol.* **77**, 802–813 (2008).

63. B. Hofner, T. Hothorn, T. Kneib, M. Schmid, A framework for unbiased model selection based on boosting. *J. Comput. Graph. Stat.* **20**, 956–971 (2011).

64. M. D. Spalding, H. E. Fox, G. R. Allen, N. Davidson, Z. A. Ferdaña, M. Finlayson, B. S. Halpern, M. A. Jorge, A. Lombana, S. A. Lourie, K. D. Martin, E. McManus, J. Molnar, C. A. Recchia, J. Robertson, Marine ecoregions of the world: A bioregionalization of coastal and shelf areas. *Bioscience* **57**, 573–583 (2007).

65. W. Cramer, R. J. Olson, S. D. Prince, J. M. O. Scurlock, in *Terrestrial Global Productivity*, J. Roy, B. Saugier, H. A. Mooney, Eds. (Academic Press, 2001), pp. 429–448.

66. J. Goudriaan, J. J. R. Groot, P. W. J. Uithol, Productivity of agro-ecosystems, in *Terrestrial Global Productivity*, J. Roy, B. Saugier, H. A. Mooney, Eds. (Academic Press, 2001), chap. 13, pp. 301–313.

67. Z. V Finkel, Marine Net Primary Production, in *Global Environmental Change*, B. Freedman, Ed. (Springer, 2014), pp. 117–124.

68. J. E. Cloern, S. Q. Foster, A. E. Kleckner, Phytoplankton primary production in the world's estuarine-coastal ecosystems. *Biogeosciences* **11**, 2477–2501 (2014).

69. M. Ateweberhan, J. H. Bruggemann, A. M. Breeman, Effects of extreme seasonality on community structure and functional group dynamics of coral reef algae in the southern Red Sea (Eritrea). *Coral Reefs* **25**, 391–406 (2006).

70. B. E. Lapointe, Nutrient thresholds for bottom-up control of macroalgal blooms on coral reefs in Jamaica and southeast Florida. *Limnol. Oceanogr.* **42**, 1119–1131 (1997).
